# The Effect of Ticagrelor for Endovascular Intervention of Intracranial Aneurysm Patients with or without Clopidogrel Resistant: A Meta-Analysis

**DOI:** 10.3390/brainsci12081077

**Published:** 2022-08-14

**Authors:** Pengfei Xia, Yimin Huang, Gang Chen

**Affiliations:** 1 Department of Neurosurgery, The First Affiliated Hospital of Soochow University, 188 Shizi Street, Suzhou 215006, China; 2 Department of Neurosurgery, Tongji Hospital, Tongji Medical College, Huazhong University of Science and Technology, Wuhan 430030, China; 3 Department of Neurosurgery & Brain and Nerve Research Laboratory, The First Affiliated Hospital of Soochow University, 188 Shizi Street, Suzhou 215006, China

**Keywords:** ticagrelor, clopidogrel, endovascular interventional, aneurysm

## Abstract

Endovascular interventional is an important treatment method for intracranial aneurysms. However, due to the risk of intracranial thrombosis, prophylactic use of antiplatelet drugs is generally required. Clinically, the most commonly used drugs are aspirin and clopidogrel; although the latter can significantly reduce the incidence of thromboembolic complications, there are still some patients with clopidogrel resistance who have ischemic adverse events during antiplatelet therapy. In this study, cohort studies of PubMed, Embase and Cochrane Library Databases were retrieved to compare the efficacy and safety of ticagrelor and clopidogrel in endovascular interventional treatment of intracranial aneurysms. A total of 10 cohort studies involving 1377 patients were included in this study. All patients had intracranial aneurysms and underwent endovascular intervention. Platelet function was measured in four of the studies and switched the patients with clopidogrel resistance to ticagrelor, while the remaining six studies did not test platelet function, but were also treated with ticagrelor or clopidogrel. The results of the study showed that ticagrelor, like clopidogrel, can effectively control thrombotic complications in endovascular interventional patients, and also control the occurrence of ischemic complications in clopidogrel-resistant patients. Ticagrelor, as a novel platelet aggregation inhibitor that can reversibly bind to P2Y12 receptor, can effectively control thromboembolic complications without increasing hemorrhagic complications, and is also effective in patients with clopidogrel resistance.

## 1. Introduction

Intracranial aneurysm is one of the most common cerebrovascular diseases, with an incidence of approximately 2–7% in adults. With the extension of human lifespan and the advances in detection methods, the prevalence of intracranial aneurysms has notably increased [1,2,3,4]. The main risk of intracranial aneurysm includes rupture and aneurysm subarachnoid hemorrhage, which has high mortality and morbidity. The overall annual rupture rate of intracranial aneurysm is about 1%, and the risk factors of aneurysm rupture include aneurysm size, aneurysm location in the posterior circulation, the pseudoaneurysm secondary to aneurysm and the previous history of subarachnoid hemorrhage [4,5,6]. The treatment targeting intracranial aneurysms is complex, and in recent years treatment regimens have become increasingly individualized. Treatment regimen decisions require a comprehensive balance between the potential rupture risk of intracranial aneurysms and the risks associated with treatment. Current treatment options for aneurysms include conservative treatment combined with imaging follow-up, surgical clipping, and endovascular intervention [7,8,9]. With the development of modern interventional technology, the advantages of intravascular treatment of intracranial aneurysm are increasingly prominent, but at the same time there is a certain risk of thrombosis; therefore, antiplatelet therapy is required and becomes necessary during the perioperative and postoperative periods [10,11].

Antiplatelet therapy is a crucial component of endovascular treatment for intracranial aneurysms. Current evidence reveals that proper preoperative and postoperative antiplatelet therapy can significantly reduce thromboembolic events. Currently, the preferred antiplatelet therapy drugs are mainly aspirin and clopidogrel. Clopidogrel is a thiophene pyridine antiplatelet drug that can inhibit platelet aggregation by generating active mercaptan metabolites under the catalysis of liver cytochrome P450 enzyme, which selectively and irreversibly bind to the P2Y12 receptor on the surface of the platelet membrane, inhibit the ADP-platelet receptor binding and the activation of the ADP-mediated glycoprotein GPIIb/IIIa complex [12]. Clopidogrel could also inhibit platelet aggregation induced by the ADP independent pathway [13].

However, certain patients will still have adverse events of stent thrombosis during long-term dual antiplatelet therapy, which reduce the expected therapeutic effect of interventional treatment as well as the benefits to patients. Previous studies believed that clopidogrel and aspirin resistance were the main causes of ischemic cerebrovascular events after interventional therapy, and clopidogrel resistance was more common [14,15]. At present, various clinical studies have proved that in the antiplatelet therapy of percutaneous coronary intervention, instead of clopidogrel, the application of ticagrelor is capable of overcoming clopidogrel resistance, reducing the occurrence of ischemic complications in the cardiac and cerebrovascular systems. And at the same time, ticagrelor application will not increase the risk of bleeding or death [16,17,18]. Ticagrelor is a new type of cyclopentyl triazolacil antiplatelet drug, which is a non-precursor drug and can directly act without liver metabolism activation. It was found to reversely bind to the P2Y12 receptor on the platelet membrane, block the signal transduction pathway and inhibit platelet aggregation [19,20]. However, there are not many studies on ticagrelor versus clopidogrel in patients with aneurysms undergoing interventional therapy. Based on existing research evidence, this study conducted a meta-analysis to determine the therapeutic value of ticagrelor for patients with aneurysms undergoing interventional therapy with or without clopidogrel resistance.

## 2. Methods

### 2.1. Search Strategy

This study was pre-defined and reported according to the preferred reporting items in the System Review and Meta-analysis (PRISMA) guidelines. A systematic literature search of MEDLINE, EMBASE, and the Cochrane Central Register of Controlled Trials (CENTRAL) from inception to 4 May 2022 was conducted by two independent investigators; the following search terms were employed: (Aneurysm [Title/Abstract]) AND (Clopidogrel OR SC 25989C OR SC 25990C OR SR 25989 OR Iscover OR PCR 4099 OR PCR-4099 OR Plavix [Title/Abstract]) AND (Ticagrelor OR Brilique OR AZD 6140 OR AZD6140 OR AZD-6140 OR Brilinta OR 3-(7-((2-(3,4-Difluorophenyl)cyclopropyl)amino)-5-(propylthio)-3H-(1-3)-triazolo(4,5-d)pyrimidin-3-yl)-5-(2-hydroxyethoxy)cyclopentane-1,2-diol [Title/Abstract]). In addition, the reference lists of relevant articles were further manually and independently searched to filter eligible studies.

### 2.2. Inclusion and Exclusion Criteria

The inclusion criteria were set as follows: (1) Study type: the study should be randomized controlled trials (RCT), a case control study or a cohort study. (2) Language restriction: English. (3) participants: patients who received intracranial aneurysms with endovascular treatment. (4) Intervention: received antiplatelet therapy with clopidogrel or ticagrelor. (5) Outcomes: reports estimate describing hemorrhagic complications or thromboembolism complications.

The exclusion criteria were set as follows: (1) Case reports, review or animal experiments. (2) Patients with intracranial aneurysms were not treated with endovascular therapy. (3) Does not report relevant outcomes on hemorrhagic complications or thromboembolism complications.

### 2.3. Study Selection and Data Extraction

The retrieved studies were further evaluated by two independent researchers based on the inclusion and exclusion criteria mentioned above; in the event of disagreement between the two researchers, the final decision was made by the third researcher. The following information was extracted from the included studies when available: first author, published year, sample size, type of aneurysm, antiplatelet treatment regimens, follow-up time, incidence of thromboembolism complications, hemorrhagic complications and neurologic disability. The risk of bias was assessed with the Cochrane Collaboration’s tool Review Manager version 5.4.1 software program (Cochrane, London, UK). In detail, a total of 87 articles were initially selected, including 26 from PubMed, 53 from Embase, and 8 from the Cochrane Library database. Of these, 23 were excluded due to duplication, 42 articles were excluded by reading title and abstract of the literature as not meeting the inclusion criteria, and 13 were excluded due to not meeting the inclusion criteria after full text reading; among them, there were 5 reviews, 5 whose valid data could not be extracted, and 3 case reports. Finally, a total of 10 articles were included. When disagreement occurred, Prof. Dr. Gang Chen made the final decision and quality control.

### 2.4. Statistical Analysis

The statistical analysis of this meta-analysis was performed using the Review Manager 5.4.1 Version software. To evaluate the effects of different antiplatelet quality regimens on hemorrhagic complications, thromboembolism complications and neurologic disability event, the effects model with Hazard Rate (HR) and its 95% confidence interval (CI) were used as evaluation indexes. In the forest map, if the rhombus graph of the combined effect size does not coincide with the invalid line, it indicates that the combined result is statistically significant, and *p* < 0.05. In addition, we performed a subgroup analysis based on whether patients tested for clopidogrel resistance. An inconsistency index statistic (I^2^) indexwas used to quantifythe degree heterogeneity among the included studies I^2^ > 50% was considered as substantial heterogeneity, if there was no statistical heterogeneity among the included studies. The fixed-effect model was used for meta-analysis, and if there was statistical heterogeneity among the results, the random-effect model was used for meta-analysis after excluding the influence of obvious clinical heterogeneity. We registered on PROSPERO with the ID number 348494.

## 3. Results

### 3.1. Study Selection

**Figure 1:**
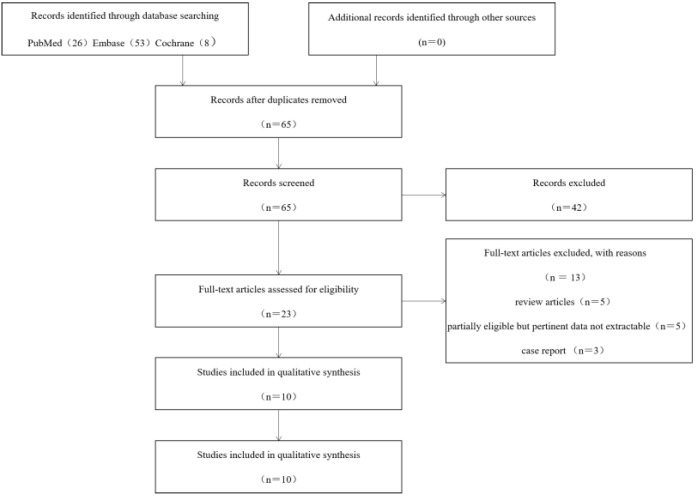
Flow diagram of the study selection process.

**Figure 2:**
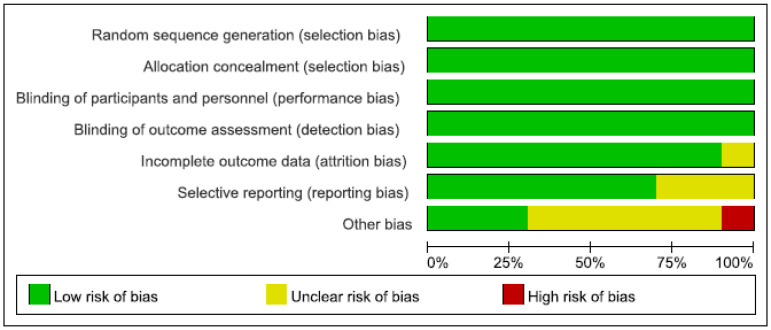
Risk of bias: a summary table for each risk of bias item for each study.

A total of 87 articles were initially retrieved, including 26 from PubMed, 53 from Embase, and 8 from the Cochrane Library database. Of these, 23 were excluded due to duplication, 42 articles were excluded by reading title and abstract of the literature as not meeting the inclusion criteria, and 13 were excluded due to not meeting the inclusion criteria after full text reading; among them, there were 5 reviews, 5 whose valid data could not be extracted, and 3 case reports. Finally, a total of 10 articles were included. Figure 1 indicates the PRISMA flow diagram. The risk of bias for the 10 studies was assessed with the domain-based Cochrane Collaboration’s tool and exhibited in Figure 2.

### 3.2. Study Baseline Characteristics

All studies were published between 2017 and 2021. Of the 10 studies, 2 [21,22] were multicenter and 8 [23,24,25,26,27,28,29,30] were single-center, involving a total of 1377 patients receiving endovascular therapies for aneurysms from six countries with sample sizes ranging from 78 to 321, including 1 [24] RCT study, 1 [23] prospective cohort study and 8 [21,22,25,26,27,28,29,30] retrospective cohort studies. Endovascular therapies contain spring coil embolization, stent assisted coil embolization and flow diverter procedures, perioperative and postoperative with clopidogrel or ticagrelor antiplatelet therapy; 4 [22,23,28,29] of the studies tested for platelet function and changed the antiplatelet regimen to ticagrelor in patients with clopidogrel resistance. In the statistics of outcomes, all studies included thromboembolism complications, 7 included tests for hemorrhagic complications, and 4 included neurologic disability. The clinical characteristics of these studies are listed in Table 1.

### 3.3. Efficacy Outcomes

#### 3.3.1. Thromboembolic Complications

**Figure 3:**
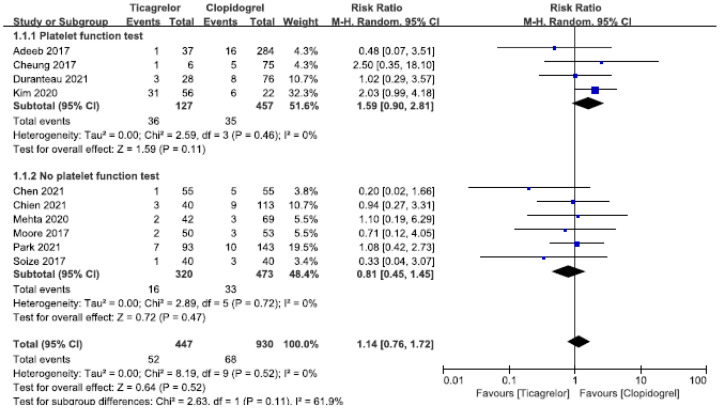
Forest plots of the meta-analysis comparing thromboembolic complications between ticagrelor and clopidogrel treatment. Top, the studies with platelet function test, and the subgroup analysis for the patients with clopidogrel resistance study. Bottom, the studies with no platelet function test.

In the meta-analysis of perioperative and postoperative application of clopidogrel or ticagrelor in patients with aneurysms after endovascular interventional surgery, 10 studies were included in the thromboembolic complications analysis; 5 [21,22,24,26,30] studies showed a decreased trend toward thromboembolic complications compared with the clopidogrel group, and 5 [23,25,27,28,29] studies showed a poorer trend. The results of the pooled 10 studies showed that, like clopidogrel, ticagrelor had a good prevention efficacy in the analysis of the efficacy of thromboembolic events; there was no significant difference (RR = 1.14, 95% CI: 0.76–1.72, *p* = 0.52). There was no significant heterogeneity across the pooled studies (I^2^ = 0%, *p* = 0.52), as shown in Figure 3.

In the subgroup analysis, all studies were divided into two groups according to whether the patients had platelet function assays, of which 4 [22,23,28,29] studies were carried out platelet function assays, and the patients of these 4 experiments divided into a clopidogrel resistance group and no clopidogrel resistance group according to the measurement results, in which clopidogrel-resistant patients were given ticagrelor in the aneurysm intravascular treatment perioperative and postoperative period. The results showed that ticagrelor in patients with clopidogrel resistance can prevent the occurrence of thromboembolic complications; the efficacy is the same as that of clopidogrel in patients without clopidogrel resistance (RR = 1.59, 95% CI: 0.90–2.81, *p* = 0.11). No heterogeneity (I^2^ = 0%, *p* = 0.46) was observed in these studies, as shown in Figure 3.

#### 3.3.2. Hemorrhagic Complications

**Figure 4:**
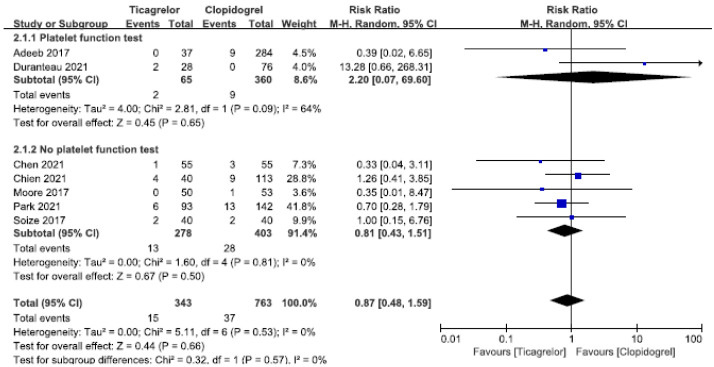
Forest plots of the meta-analysis comparing hemorrhagic complications between ticagrelor and clopidogrel treatment. Top, the studies with platelet function test, and the subgroup analysis for the patients with clopidogrel resistance study. Bottom, the studies with no platelet function test.

A total of 7 [22,24,26,27,28,30] studies were conducted to monitor hemorrhagic complications, and 2 [26,28] of them showed that ticagrelor had a tendency to cause bleeding complications compared with clopidogrel, but the results of 5 [21,22,24,27,30] studies were opposite, and there was no significant statistical difference. Meta-analysis of the 7 studies did not show that ticagrelor increased the incidence of perioperative and postoperative hemorrhagic complications after endovascular treatment of aneurysms compared with clopidogrel (RR = 0.87, 95% CI: 0.48–1.59, *p* = 0.66). NO heterogeneity (I^2^ = 0%, *p* = 0.53) was observed in these studies, as shown in Figure 4.

Subgroup analysis showed that only 2 [22,28] studies simultaneously measured platelet function and monitored hemorrhagic complications. Ticagrelor did not increase the incidence of hemorrhagic complications in patients with clopidogrel resistance (RR = 2.20, 95% CI: 0.07–69.60, *p* = 0.66), but the results were highly heterogeneous (I^2^ = 64%, *p* = 0.09), possibly due to the fact that there were too few studies included, as shown in Figure 4.

#### 3.3.3. Neurologic Disability

**Figure 5:**
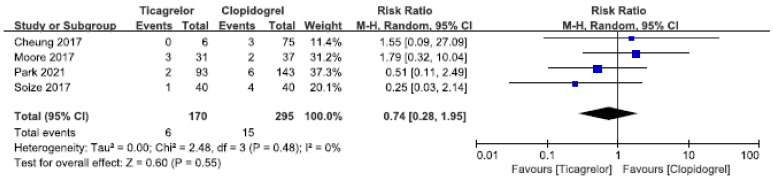
Forest plots of the meta-analysis comparing Neurologic disability between ticagrelor and clopidogrel treatment.

A total of 4 [21,27,29,30] studies were included in the study of neurologic disability, among which 1 [29] study tested platelet function; although ticagrelor showed a good trend in neurological function recovery, the difference was not significant. Overall meta-analysis showed that ticagrelor had the same efficacy as clopidogrel in neurologic disability events (RR = 0.74, 95% CI: 0.28–1.95, *p* = 0.55). NO heterogeneity (I^2^ = 0%, *p* = 0.48) was observed in these studies, as shown in Figure 5.

#### 3.3.4. Sensitivity Analysis and Publication Bias Evaluation

Sensitivity analysis was performed on each outcome indicator of the ticagrelor group and the clopidogrel group, and the studies were removed one by one to recombine the data; the effect model of these meta-analysis results did not change, suggesting that the results were stable. Publication bias was detected based on the outcomes of ticagrelor and clopidogrel groups. Due to the limited number of included studies, Egger’s test was performed on Stata16.0 software; the results show there was no significant publication bias between the studies. Hemorrhagic complications (*p* = 0.910); thromboembolism complications; platelet function test subgroup (*p* = 0.416); no platelet function test subgroup (*p* = 0.065); neurologic disability (*p* = 0.881).

## 4. Discussion

With the advances in technology, the treatment of intracranial aneurysm has made remarkable progress. Intracranial aneurysm interventional embolization has become one of the main treatment methods. Its characteristics of small trauma, quick recovery and reliable efficacy play a significant role in accelerating the rehabilitation and improving the prognosis of patients. At present, the main intravascular interventional therapy methods include coiling alone, balloon-assisted coiling, stent-assisted coiling and a blood-flow guidance device. Compared with simple embolization and balloon-assisted embolization, stent-assisted embolization technology can greatly improve the indications for the treatment of cerebral aneurysm and reduce the recurrence rate. However, the incidence of perioperative thromboembolic events in patients with stent-assisted aneurysm embolization is higher than that in patients with simple embolization and balloon-assisted aneurysm embolization, making it necessary for them to take dual antiplatelet drugs for a long time, or even lifelong antiplatelet therapy [31]. As a new type of dense mesh stent, the flow diverter procedure has the characteristics of high metal coverage and a small mesh, which can change the blood flow function in the parent artery and promote the repair of the vascular intima at the neck of the aneurysm. Its indications mainly include large aneurysms, wide-necked aneurysms, fusiform aneurysms and dissecting aneurysms, but flow diverter procedures can also increase the embolization rate of aneurysms [32]; therefore, like stenting, the flow diverter procedure also requires long-term antiplatelet therapy.

Clopidogrel and aspirin are the most commonly used antiplatelet drugs, which can significantly reduce the incidence of thrombotic complications after intracranial aneurysm intervention. However, some patients still have ischemic adverse events during the use of clopidogrel and aspirin double antiplatelet therapy; the fact that clopidogrel does not respond to some people is the main reason for the ineffectiveness of antiplatelet therapy, while the polymorphism of clopidogrel metabolic enzyme genes may be one of the factors affecting the antiplatelet effect, mainly including CYP2C19, ABCB1, P2RY12 and CES1, etc. [33,34]. Therefore, some studies suggest that the platelet function should be evaluated before intervention, such as the P2Y12 reactive units; the inhibition rate of platelet function should reach over 30%, otherwise clopidogrel needs to be replaced with other antiplatelet drugs to achieve the standard of the platelet function inhibition rate [35].

In the process of antiplatelet aggregation, clopidogrel binding to the P2Y12 receptor is irreversible, which may increase the risk of bleeding complications during medication. Some clinical studies have therefore adopted ticagrelor that can bind reversibly to P2Y12 receptors to improve prognosis [36,37]. In addition, the polymorphism of the ticagrelor CYP2C19 gene has no significant effect on the active ingredient of the drug, and drug resistance should not occur. These suggest that ticagrelor is a precursor drug that can act on platelet receptors and effectively inhibit platelet aggregation with high safety.

The results of this meta-analysis showed that ticagrelor had the same efficacy as clopidogrel in 6 cohort studies of interventional aneurysm therapy without platelet function test, and could effectively control patients’ thrombosis complications without increasing the incidence of hemorrhagic complications. In 4 studies with clopidogrel resistance, ticagrelor also reduced the incidence of thrombotic complications as effectively as clopidogrel did in patients without clopidogrel resistance in platelet function. In addition, a number of single-arm trials have demonstrated the efficacy of ticagrelor in antiplatelet therapy after stenting and flow diverter procedures [37,38,39]. Studies have found that ticagrelor did not increase neurological complications in patients undergoing intervention for unruptured aneurysms compared with clopidogrel [40]. Besides, ticagrelor alone as a single antiplatelet agent is also safe and effective in the flow diversion therapy of complex aneurysms [41].

However, this meta-analysis also has limitations that cannot be ignored. First, our data were based on 10 limited clinical studies, only 4 of which measured platelet function and compared the efficacy of ticagrelor. Although heterogeneity was not found in most outcomes, there was a high level of heterogeneity in hemorrhagic complications, which may be due to the small number of studies included. Further studies would be needed for comprehensive analysis regarding hemorrhagic complications. Additionally, in this study, we have set the inclusion/exclusion criteria, in which age, gender, and area of study are not considered. However, case reports, review and animal experiments were excluded. Secondly, most of the studies included in this paper were retrospective studies, and only one was a randomized controlled study, which may also be the reason for the heterogeneity among the studies. In addition, some studies provided too short follow-up results, among which the shortest follow-up time was only 1 month.

## 5. Conclusions

In summary, ticagrelor can effectively control thromboembolism complications without increasing hemorrhagic complications during perioperative and postoperative interventional aneurysm therapy, and its efficacy is also significant in clopidogrel-resistant patients. However, there are still limitations in this meta-analysis, and the conclusions of this study still need more relevant clinical studies with a reasonable design, rigorous execution and a large sample size to further verify.

## Figures and Tables

**Table 1:** Characteristics of studies included in the meta-analysis.

Study	Year	Cases	Type of Aneurysm	Antiplatelet Drug	Follow-Up Time	Hemorrhage	Thromboembolism	Neurologic Disability
Moore	2017	103	Unruptured + ruptured	ASA + ticagrelor 2*90 mg/d (50)	7 m	0	2	3
				ASA + clopidogrel 75 mg/d (53)		1	3	2
Soize	2017	80	unruptured	ASA + ticagrelor 2*90 mg/d (40)	3 m	2	1	1
				ASA+clopidogrel 75 mg/d (40)		2	3	4
Kim	2020	78	unruptured	ticagrelor 2*90 mg/d (56)	3 m	N/A	31	N/A
				ASA + clopidogrel 75 mg/d (22)		N/A	6	N/A
Park	2021	235	unruptured	ASA 81 mg/d + ticagrelor 2*90 mg/d (93)	3 m	6	7	2
				ASA 81 mg/d + clopidogrel 75 mg/d (142)		13	10	6
Cheung	2017	81	N/A	Ticagrelor (6)	12 m	N/A	1	0
				clopidogrel (75)		N/A	5	3
Duranteau	2021	104	N/A	ASA + ticagrelor 2*90 mg/d (28)	1 m	2	3	N/A
				ASA + clopidogrel 75 mg/d (76)		0	8	N/A
Chen	2021	110	N/A	ASA 100 mg/d + ticagrelor 2*90 mg/d (55)	12 m	1	1	N/A
				ASA 100 mg/d + clopidogrel 75 mg/d (55)		3	5	N/A
Mehta	2020	111	unruptured	ASA 81 mg/d + ticagrelor 2*45 mg/d (42)	12 m	N/A	2	N/A
				ASA 325 mg/d + clopidogrel 75 mg/d (69)		N/A	3	N/A
Chien	2021	153	unruptured	ASA 75 mg/d + ticagrelor 2*90 mg/d (40)	12 m	4	3	N/A
				ASA 75 mg/d + clopidogrel 75 mg/d (113)		9	9	N/A
Adeeb	2017	321	Unruptured + ruptured	ASA + ticagrelor (37)	13 m	0	1	N/A
				ASA + clopidogrel 75 mg/d (284)		9	16	N/A

ASA; Aspirin, m: mouth, N/A: not applicable.
